# Disordered oropharyngeal microbial communities in H7N9 patients with or without secondary bacterial lung infection

**DOI:** 10.1038/emi.2017.101

**Published:** 2017-12-20

**Authors:** Hai-feng Lu, Ang Li, Ting Zhang, Zhi-gang Ren, Kang-xin He, Hua Zhang, Jie-zuan Yang, Qi-xia Luo, Kai Zhou, Chun-lei Chen, Xia-liang Chen, Zhong-wen Wu, Lan-juan Li

**Affiliations:** 1 State Key Laboratory for Diagnosis and Treatment of Infectious Diseases, Collaborative Innovation Center for Diagnosis and Treatment of Infectious Diseases, The First Affiliated Hospital, College of Medicine, Zhejiang University, Hangzhou 310003, China; 2 Center of Precision Medicine, The First Affiliated Hospital of Zhengzhou University, Zhengzhou 450052, China; 3 Department of Hematology, The First Affiliated Hospital, School of Medicine, Zhejiang University, Hangzhou 310003, China; 4 Department of Traditional Chinese Medicine, The First Affiliated Hospital, School of Medicine, Zhejiang University, Hangzhou 310003, China

**Keywords:** H7N9, microbiota-targeted prophylactic therapies, oropharyngeal microbiome, secondary bacterial lung infection

## Abstract

Secondary bacterial lung infection (SBLI) is a serious complication in patients with H7N9 virus infection, and increases disease severity. The oropharyngeal (OP) microbiome helps prevent colonisation of respiratory pathogens. We aimed to investigate the OP microbiome of H7N9 patients with/without secondary bacterial pneumonia using 16S rRNA gene sequencing. OP swab samples were collected from 51 H7N9 patients (21 with SBLI and 30 without) and 30 matched healthy controls (HCs) and used for comparative composition, diversity and richness analyses of microbial communities. Principal coordinates analysis successfully distinguished between the OP microbiomes of H7N9 patients and healthy subjects, and the OP microbiome diversity of patients with SBLI was significantly increased. There was significant dysbiosis of the OP microbiome in H7N9 patients, with an abundance of *Leptotrichia*, *Oribacterium*, *Streptococcus*, *Atopobium*, *Eubacterium*, *Solobacterium* and *Rothia* species in patients with SBLI, and *Filifactor*, *Megasphaera* and *Leptotrichia* species in patients without SBLI, when compared with HCs. Importantly, *Haemophilus* and *Bacteroides* species were enriched in HCs. These findings revealed dysbiosis of the OP microbiota in H7N9 patients, and identified OP microbial risk indicators of SBLI, suggesting that the OP microbiome could provide novel and non-invasive diagnostic biomarkers for early microbiota-targeted prophylactic therapies for SBLI prevention.

## INTRODUCTION

During the avian influenza A (H7N9) epidemics of the last 3 years, ~150 cases were confirmed in Zhejiang Province, China.^1^ Similar to the highly pathogenic H5N1 strain, H7N9 causes severe respiratory distress syndrome in most patients. However, because of clinical practice experience, findings from ongoing research and intensive care management, it has a lower case-fatality rate than H5N1.^2^ Despite this, the incidence of secondary bacterial pneumonia, which is mainly caused by multidrug-resistant *Acinetobacter baumannii* and *Klebsiella pneumoniae*, is much higher.^3^ Secondary bacterial lung infection (SBLI) is a particularly serious complication of influenza infection.^4, 5^ Current treatment strategies are based on routine culture-based diagnosis methods. Recent studies have highlighted the clinical significance of influenza-associated bacterial pneumonia, but most have focused directly on the host immune response,^4^ and have not examined changes in the human airway microbiota that lead to heightened susceptibility to subsequent pathogenic infections. When in equilibrium, the airway microbiome can restrict the growth of multiple invading pathogens.^6^ Disruption of the microbial balance, which can result from acquisition of viral pathogens or through immunological perturbations, can have adverse consequences. These include pathogen overgrowth and dissemination, which can lead to symptomatic infections such as pneumonia.^7^ Therefore, an urgent investigation of variations in the airway microbiota of H7N9 patients with and without SBLI is needed.

Studying variations in the lung microbiota of H7N9 patients is challenging because lung microbiome sampling is an invasive surgical procedure that may cause harm to the subjects.The oropharynx is colonised by various important bacterial species, most of which are commensal and required to maintain the health of the airway. The oropharyngeal (OP) microbiome therefore serves as a gatekeeper of the airway, providing resistance against colonisation by respiratory pathogens. Dysbiosis between regular residents of the OP microbiome is involved in pathogen overgrowth, and consequently lung disease.^8^ Moreover, it is easily sampled, with minimal disturbance of the existing microbiome, and there is little risk or discomfort to participants. A more detailed understanding of significant variations in the OP microbiome in disease states and preclinical conditions may provide greater insight into the pathogenesis of pneumonia, and a better understanding of disease onset and progression.

Herein, we characterised the OP microbiome of H7N9 patients with and without SBLI, along with healthy controls (HCs), using next-generation sequencing analysis of the bacterial 16S rRNA gene.^9^ We aimed to examine the association between dysbiosis of the OP microbiota and avian influenza A (H7N9) virus infection, and assess the susceptibility of these patients to SBLI during their hospitalisation. We anticipate that these results will be helpful for early microbiota-targeted prophylactic therapies for SBLI in H7N9 patients.

## MATERIALS AND METHODS

### Subjects and sampling

The study was conducted between 25 March 2014 and 31 March 2016 at the First Affiliated Hospital, College of Medicine of Zhejiang University, Hangzhou, China. Approval was obtained from the ethical board of the hospital (reference number: 2013-157). Written informed consent and questionnaires addressing previous and current diseases, lifestyles and medication (Supplementary Table S1) were obtained from all subjects who voluntarily provided samples.

All volunteers with suspected H7N9 infection were enrolled in the study by their physicians, and provided OP swab and nasal lavage fluid samples at fever clinics at our hospital if they fulfilled the following criteria: (i) aged between 50 and 68 years with no history of smoking or alcohol abuse; (ii) clinical symptoms consistent with acute influenza (fever, cough, coryza and difficulty breathing); and a positive rapid test for influenza A using an influenza A virus antigen screening test kit according to the standard protocols (Kehua Bio-engineering Co., Shanghai, China); (iii) had episodes of other airway disease, such as asthmatic bronchitis, or were immunosuppressed in the 6 months before hospitalisation; and (iv) had normal oral mucous membranes and were free from non-restored carious lesions.

To avoid factors that may provoke an alteration in the OP microbiome, such as medical intervention, the OP swab samples were taken from each enrolled subject as close as possible to the time of enrolment, and were collected before nasal lavage fluid sampling. Nasal lavage fluid samples were transferred to the clinical gene amplification testing laboratory at our hospital, and RNA was extracted with the use of the QIAamp Viral RNA Mini Kit (Qiagen, Hilden, Germany), according to the manufacturer’s instructions, Real-time reverse transcription-PCR assays for detecting H7N9 virus as described previously.^10^ OP samples were collected and pretreated as previous research.^11^

The OP swab samples were discarded if (i) they produced a negative result from PCR-based H7N9 testing, (ii) the patient had used antibiotics in the month before hospitalisation, (iii) the patient contracted a SBLI after receiving artificial respiration intubation surgery or antibiotic treatment during hospitalisation and treatment in H7N9 isolation wards.

Patient data, including H7N9 PCR testing results, presence of co-morbidities (SBLI), clinical presentation and course, were obtained from medical records and laboratory information systems. Matched healthy volunteers were recruited from amongst the spouses of the patients. Healthy volunteers were within the normal ranges upon physical examination, had no history of smoking or alcohol abuse, had no airway infection, runny nose, sputum production or other systemic disease, and had not received antibiotics, probiotics or prebiotics in the month before enrolment.

In total, 81 OP swab samples were used for subsequent analysis, and classified into three groups: H7N9_SBLI (SBLI occurred during hospitalisation in H7N9 isolation wards, *n*=21); H7N9 (no SBLI, *n*=30); HC (healthy volunteers, *n*=30).

### DNA extraction

Microbial DNA was extracted from the OP samples using a Qiagen Mini DNA Extraction Kit (Qiagen) with a modified protocol for cell lysis. Briefly, the pellet was resuspended in 400 μL of ASL (included in the kit). Acid-washed glass beads (100 mg; diameter <0.1 mm; Sigma Chemical Co., St. Louis, MO, USA) were added to the suspension, and the mixture was vortexed vigorously for 45 s, and then incubated for 5 min at 95 °C. The remainder of the extraction procedure was carried out as per the manufacturer’s protocol. The DNA was finally eluted twice in 30 μL of TE buffer (10 mM Tris, 1 mM EDTA, pH 8), and quantified using a Qubit 2.0 Fluorometer (Invitrogen, Carlsbad, CA, USA).

### PCR and sequencing

The protocols for V3-V4 amplification and sequencing strategy were described in our previous study.^12^ The raw reads were deposited into the European Nucleotide Archive database (study accession no. PRJEB 20509).

### Sequence assembly and analysis

Sequence assembly and analysis was carried out as described previously.^12^ The size of each sample was equalised by random subtraction to 4000 reads. The remaining sequences were binned into operational taxonomic units (OTUs) using USEARCH software, with a cutoff of 97% identity.^13^ For each OTU, reads present at the highest frequencies were chosen as representative sequences.

Representative sequences were assigned at different taxonomic levels (from phylum to genus) to the bacterial SILVA data set following the Bayesian approach, with a 97% cutoff value.^14^ Bacterial diversity was determined using sampling-based analysis of OTUs, and was displayed as a rarefaction curve. Bacterial richness and diversity across the samples were calculated using the following indices: Chao 1; Obs; incidence-based coverage estimators; Simpson; inverse Simpson; and Shannon, which were estimated at a distance of 3%. Principal component analysis (PCA) using weighted and unweighted UniFrac distance matrices^15^ was used to visualise the interactions among the bacterial communities of different samples. To reduce the possibility of PCA calculation errors, we used three different calculation methods, including the Hellinger distance method, Jensen–Shannon divergence analysis and the Spearman coefficient distance method.^12^ The distance analyses were conducted using a custom R programme function provided by the European Molecular Biology Laboratory (http://enterotype.embl.de/enterotypes.html#dm). The Linear Discriminant Analysis Effect Size programme (LEfSe; http://huttenhower.sph.harvard.edu/galaxy/) was used to identify taxa that differed consistently between sample types, as described previously.^16^ LEfSe was used to identify biomarkers within the OP microbiomes of both patient groups and those of HCs at multiple levels in data sets, grade the biomarker according to statistical significance and visualise the results using taxonomic bar charts and cladograms.^17^ Gene family abundance in OP microbiomes was predicted using phylogenetic investigation of communities by reconstruction of unobserved states (PICRUSt) software.^18^

### Statistical analysis

GraphPad Prism V.6.0 (GraphPad Software Inc., San Diego, CA, USA) was used for all analyses and preparation of graphs. The results of OP microbiome diversity indices were expressed as the median value, and analysed using the non-parametric Kruskal–Wallis *H*-test. Relative abundance of a microbe in a sample was calculated as the read count normalised against the total reads in that sample. This measurement was adjusted for the different sequencing read yields in the different samples. The relative abundance value for each genus was depicted as mean±sem, and statistical analyses were performed using a two-tailed non-parametric Kruskal–Wallis *H*-test (R software, Vienna, Austria: Kruskal. test) to evaluate the significance of differences in microbial taxa between groups and clinical measures. Differences with a *P*-value of <0.05 were considered significant. The Wilcoxon rank sum test was used to compare the crucial taxa between groups. To identify key genera in the OP microbiome of SBLI patients, a receiver-operating characteristic (ROC) curve for each crucial taxon was generated. The area under the parametric curve (AUC) was computed by numerical integration using the R software pROC package (10 000 bootstrap replicates) according to the protocols established in our previous study.^12^

## RESULTS

### Clinical characteristics of the study population

All patients received oseltamivir antiviral treatment according to the standard of treatment for H7N9 infection^19^ during hospitalisation in H7N9 isolation wards. In total, 21 patients developed SBLI (H7N9_SBLI group) without receiving any invasive surgery. According to their medical records, these patients had not received any antibiotic treatment before developing SBLI. Exogenous respiratory pathogens such as *Pseudomonas*, multidrug-resistant *A. baumannii* and *K. pneumonia* were isolated from sputum samples, while *Flavobacterium indologenes* and *Staphylococcus* species were isolated from venous blood samples collected 5–7 days after the onset of symptoms. Among the samples, three showed mixed bacterial lung infection, while nine indicated mixed bacterial and fungal lung infection. Two of the positive blood cultures contained *F. indologenes* and four indicated *Staphylococcus* species.

Spouses of the patients were used as HCs in an attempt to minimise the effects of living conditions, such as diet and environment, on the OP microbiome. In addition, exclusion criteria included factors such as smoking and alcohol abuse, which are reported to influence the upper airway microbiome.^20^ Therefore, after applying the strict inclusion and exclusion criteria, 81 swab samples were used for subsequent microbiome analysis. Characteristics of the study population are summarised in Table 1.

### The H7N9_SBLI group showed increased microbial diversity

**Figure 1:**
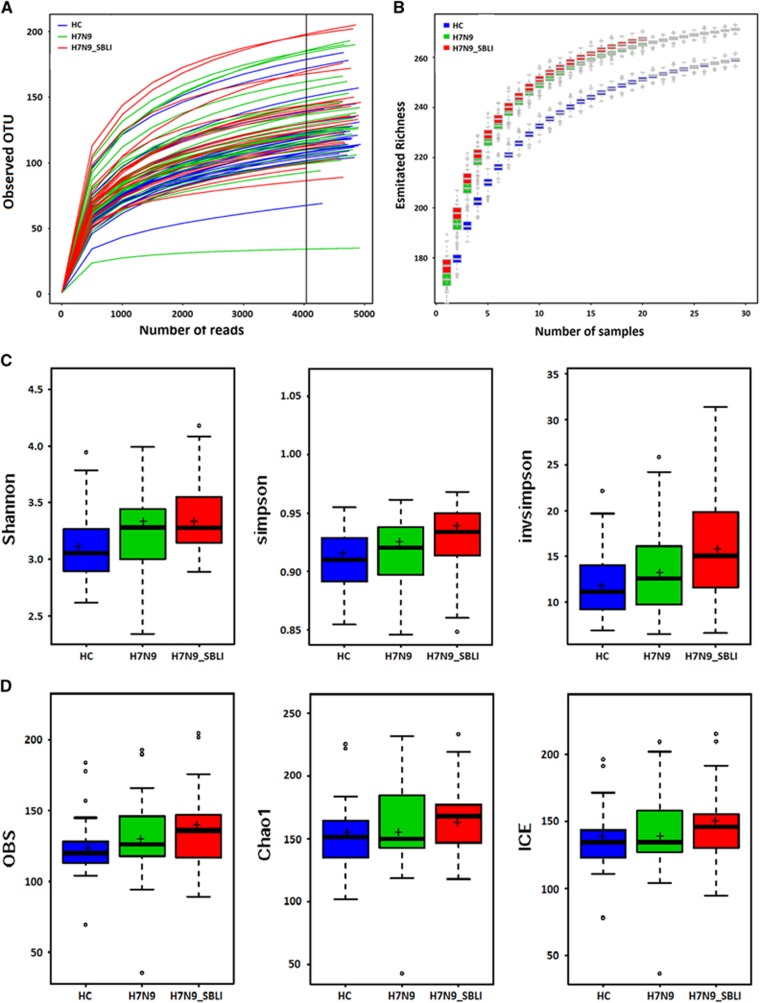
Phylogenetic diversity of oropharyngeal (OP) microbiomes among individuals and between H7N9 patients and healthy subjects. (**A**) Rarefaction analysis of bacterial 16S rRNA gene sequences was used to evaluate whether further sequencing would likely detect additional taxa, indicated by a plateau. The y axis denotes the number of operational taxonomic units detected by Miseq sequencing at the corresponding sequencing depths shown along the x axis. The subject group is indicated by the colour key at the top left corner. (**B**) Richness index curves evaluating the number of samples likely required to identify additional taxa indicated by a plateau. The y axis denotes the richness detected by Miseq sequencing at the corresponding number of samples shown along the x axis. The subject group is indicated by the colour key at the top left corner. (**C**) Box plots depict microbiome diversity differences according to the Shannon index, Simpson index and inverse Simpson index between both patient groups and the HC group. (**D**) Box plots depict microbiome diversity differences according to the Obs index, Chao 1 index and incidence-based coverage estimators index between both patient groups and the HC group. The ‘+’ symbol represents the median value, and the upper and lower ranges of the box represent the 75% and 25% quartiles, respectively. OP microbiomes of H7N9 patients with SBLI, H7N9_SBLI; OP microbiomes of patients with H7N9 virus infection, H7N9; OP microbiomes of the healthy control group, HC.

**Figure 2:**
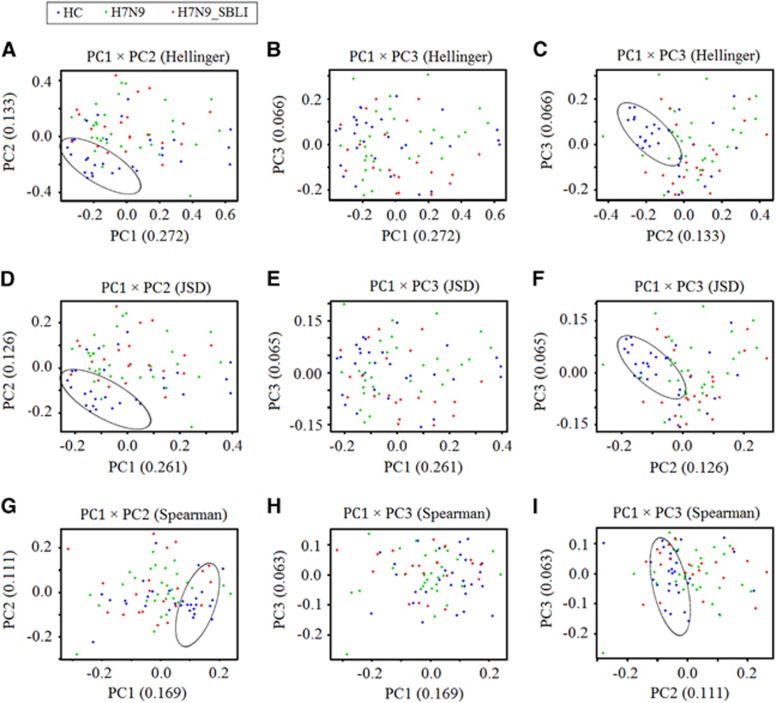
Principal coordinates analysis (PCoA) using unweighted UniFrac scores for microbiomes of H7N9 (green), H7N9 secondary bacterial lung infection (H7N9_SBLI) (red) and health control (HC) (blue) samples using different methods for calculating distances. (**A**–**C**) Hellinger distance, (**D**–**F**) Jensen–Shannon divergence analysis and (**G**–**I**) the Spearman coefficient distance. Each symbol represents a sample. The variance explained by the PCoA is indicated in parentheses on the axes (circles highlight the clustering of the oropharyngeal microbiomes of the HC group).

A total of 607 990 filtered, high-quality partial reads were generated, with a mean of 7506 reads per sample (Supplementary Data Set S1A). Rarefaction curves of numbers of observed OTUs per sample showed that the mean number of observed OTUs reached a plateau at ~4000 sequence reads (Figure 1A), and the rarefaction curves of the richness index curves per sample for each of the three cohorts also plateaued (Figure 1B). This indicated that almost all OTUs present in each group were detected, and that 4000 reads were sufficient to identify most of the bacterial community members within each OP swab microbiome. Thus, the OTU pool was randomly subsampled at 4000 reads per sample for subsequent community composition analyses. The different diversity indices indicated that the diversity of the H7N9_SBLI group OP microbiomes was significantly increased compared with that of the HC microbiomes (*P*<0.05 for all biodiversity parameters; Figure 1C), while for the H7N9 group, only two diversity indices (Shannon and Obs) were higher than the HC group. No significant difference in diversity was observed between the H7N9 and H7N9_SBLI groups. Similar to the PCA results, the unweighted (quantitative; Supplementary Figure S1A) and weighted (qualitative; Supplementary Figure S1B) UniFrac PCA plots failed to distinguish between the OP microbiomes of the H7N9 and H7N9_SBLI groups, but did distinguish the H7N9_SBLI group from the HC. Notably, the results of principal coordinates analysis using the Hellinger distance, Jensen–Shannon divergence analysis and Spearman coefficient distance methods showed that the OP microbiomes of most patient differed from those of the HC using the first and second components (Figures 2A, 2D and 2G), and using the first and third components (Figures 2C, 2F and 2I). Species richness and diversity estimates were obtained for each microbiome (Supplementary Data Set S1A).

### Bacterial taxonomic differences between patient groups and healthy subjects

**Figure 3:**
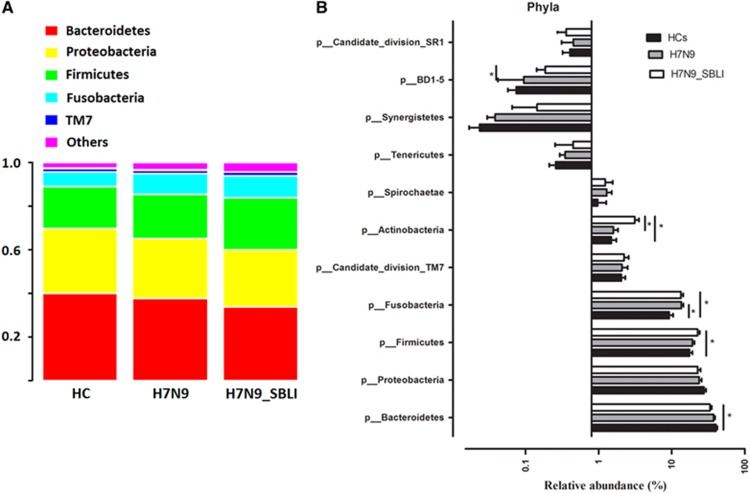
Comparison of phyla within the oropharyngeal (OP) microbiomes of the patient and healthy control (HC) groups. (**A**) Comparison of the average abundance of each bacterial phylum in each of the patient and HC groups, respectively. (**B**) Significant differences in the abundance of predominant phyla between the patient and the HC groups.The average abundance values for each bacterium is depicted as mean ±sem. *P*-values were calculated using the non-parametric Mann–Whitney test, and are shown in Supplementary Table S2. Significant differences are indicated by *P<0.05. OP microbiomes of H7N9 patients with secondary bacterial lung infection, H7N9_SBLI; OP microbiomes of patients with H7N9 virus infection, H7N9. The subject group is indicated by the colour key at the top right corner.

Using 97% as the similarity cutoff, 113 qualified genus-level OTUs were delineated (Supplementary Data Set S1_B). Five predominant phyla were represented in the OP microbial profiles: Bacteroidetes (37.6% of all reads); Proteobacteria (24.8%); Firmicutes (19.4%); Fusobacteria (11.7%); and the candidate divisions TM7 (2.1%). The remaining phyla were present at much lower relative abundances (<2% of the total reads; Figure 3A). Analysis at the phylum level showed that the relative abundance of Fusobacteria was significantly higher in both the H7N9 and H7N9_SBLI groups compared with the HC group (*P*=0.003 and 0.008, respectively, by Kruskal–Wallis test). Actinobacteria were significantly enriched in the H7N9_SBLI group compared with the H7N9 and HC groups (*P*=0.001 and 0.003, respectively, by Kruskal–Wallis test). Of the other phyla, the relative abundance of Firmicutes and Bacteroidetes was significantly increased (*P*=0.005) and decreased (*P*=0.013), respectively, in the H7N9_SBLI group compared with the HC group (Figure 3B and Supplementary Table S2).

**Figure 4:**
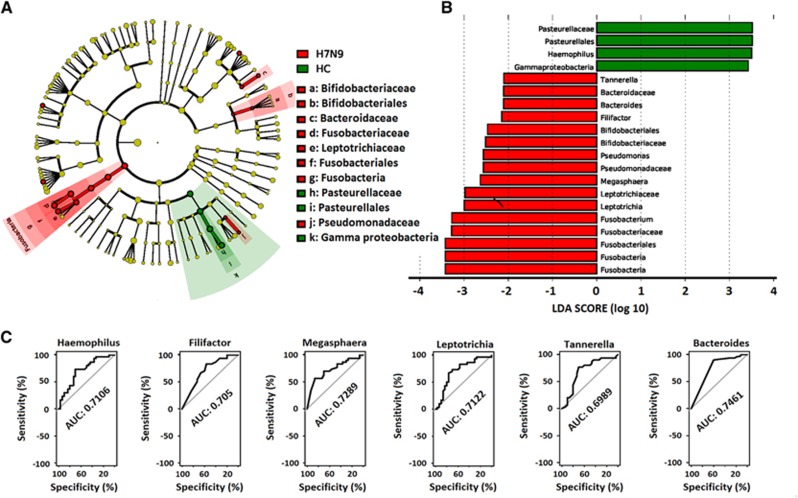
Linear discriminant analysis effect size (LEfSe) and linear discriminant analysis (LDA) based on operational taxonomic units was used to differentiate between the oropharyngeal (OP) microbiomes of the H7N9 patients and the healthy controls (HC). (**A**) Cladogram generated using the LEfSe method indicating the phylogenetic distribution of OP microbes associated with H7N9 patients (red) and healthy subjects (green). (**B**) LDA scores indicate significant differences in the microbiota between the H7N9 patients and HC. (**C**) Prediction of the key genera in the OP microbiomes of H7N9 patients and HC. Receiver-operating characteristic (ROC) plot for *Bacteroides*, area under the parametric curve (AUC) value=0.7461. ROC plot for *Megasphaera*, AUC=0.7289. ROC plot for *Leptotrichia*, AUC=0.7122. ROC plot for *Haemophilus*, AUC=0.7106. ROC plot for *Filifactor*, AUC=0.705. H7N9, OP microbiomes of patients with H7N9 virus infection.

**Figure 5:**
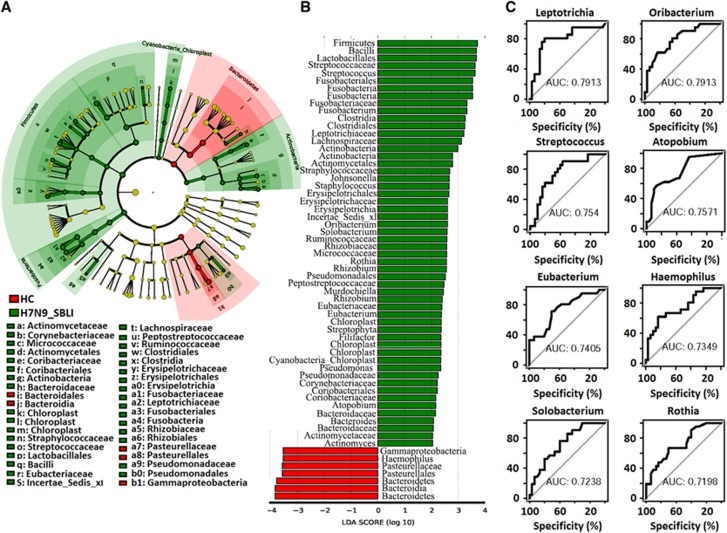
Linear discriminant analysis effect size (LEfSe) and linear discriminant analysis (LDA) based on operational taxonomic units were used to characterise differences between the oropharyngeal (OP) microbiomes of the H7N9 secondary bacterial infection (H7N9_SBLI) patients and healthy controls (HCs). (**A**) Cladogram generated using the LEfSe method indicating the phylogenetic distribution of OP microbes associated with H7N9_SBLI patients (green) and healthy subjects (red). (**B**) LDA scores indicate significant differences in the microbiota between the H7N9_SBLI patients and HCs. (**C**) Prediction of the key genera in the OP microbiomes of H7N9_SBLI patients and HC. Receiver-operating characteristic (ROC) plot for *Leptotrichia*, area under the parametric curve (AUC) value=0.7913. ROC plot for *Oribacterium*, AUC=0.7913. ROC plot for *Streptococcus*, AUC=0.754. ROC plot for *Atopobium*, AUC=0.7571. ROC plot for *Eubacterium*, AUC=0.7405. ROC plot for *Haemophilus*, AUC=0.7349. ROC plot for *Solobacterium*, AUC=0.7238. ROC plot for *Rothia*, AUC=0.7198.

**Figure 6:**
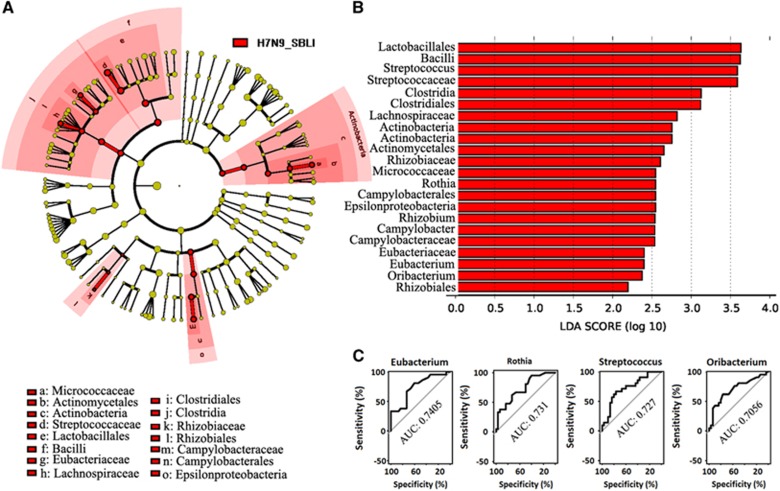
Linear discriminant analysis effect size (LEfSe) and linear discriminant analysis (LDA) based on operational taxonomic units were used to characterise differences between the oropharyngeal (OP) microbiomes of the H7N9 and H7N9 secondary bacterial infection (H7N9_SBLI) patients. (**A**) Cladogram generated using the LEfSe method indicating the phylogenetic distribution of the OP microbiota associated with H7N9_SBLI patients (red). (**B**) LDA scores indicate significant differences in the microbiota between the H7N9_SBLI patients and HCs. (**C**) Prediction of the key genera in the OP microbiomes of the H7N9_SBLI and H7N9 patients. Receiver-operating characteristic (ROC) plot for *Eubacterium*, area under the parametric curve (AUC) value=0.7405. ROC plot for *Rothia*, AUC=0.731. ROC plot for *Streptococcus*, AUC=0.727. ROC plot for *Oribacterium*, AUC=0.7056. OP microbiomes of patients with H7N9 virus infection, H7N9.

LEfSe analysis was used to compare the estimated OP microbiome phylotypes of the patient groups and the HCs. The OP microbiomes of the H7N9 group were characterised by a preponderance of Fusobactericeae, Bifidobacteriaceae, Pseudomonadaceae and Bacteroidaceae, whereas the HC microbiomes were dominated by Gammaproteobacteria (Figures 4A and 4B, and Supplementary Data Set S2A). Five key genera (*Bacteroides, Megasphaera, Leptotrichia, Haemophilus and Filifactor*) showed a significant difference in abundance between the H7N9 and HC groups (*P*<0.005), with ROC-plot AUC values of 0.746, 0.729, 0.712, 0.711 and 0.705, respectively (Figure 4C). The OP microbiomes of the H7N9_SBLI group were characterised by a preponderance of Bacteriodaceae, Actinomycetaceae, Corynebacteriaceae, Micrococcaceae, Coribacteriaceae, Straphylococcaceae, Streptococcaceae, Eubacteriaceae, Lachnospiraceae, Peptostreptococcaceae, Fusobacteriaceae, Leptotrichiaceae, Pseudomonadaceae and Rhizobiaceae, whereas the HC microbiomes were characterised by a preponderance of Pasteurellaceae, which belong to the Gammaproteobacteria class (Figures 5A and 5B, and Supplementary Data Set S2B). Eight key genera (*Leptotrichia*, *Oribacterium*, *Streptococcus*, *Atopobium*, *Eubacterium*, *Haemophilus*, *Solobacterium* and *Rothia*) showed a significant difference in abundance between the H7N9_SBLI and HC groups (*P*<0.005), with ROC-plot AUC values of 0.791, 0.791, 0.754, 0.757, 0.741, 0.735, 0.724 and 0.720, respectively (Figure 5C). The OP microbiomes of the H7N9_SBLI group showed more severe dysbiosis than the H7N9 group, which was reflected in the greater abundance of Micrococcaceae, Streptococcaceae, Eubacteriaceae, Lachnospiraceae, Rhizobiaceae and Campylobacteraceae bacteria in these swab samples (Figures 6A and 6B, and Supplementary Data Set S2C). In addition, four key genera (*Eubacterium*, *Rothia*, *Streptococcus* and *Oribacterium*) showed a significant difference in abundance between the H7N9_SBLI and H7N9 groups (*P*<0.01), with ROC-plot AUC values of 0.741, 0.731, 0.727 and 0.706, respectively (Figure 6C).

**Figure 7:**
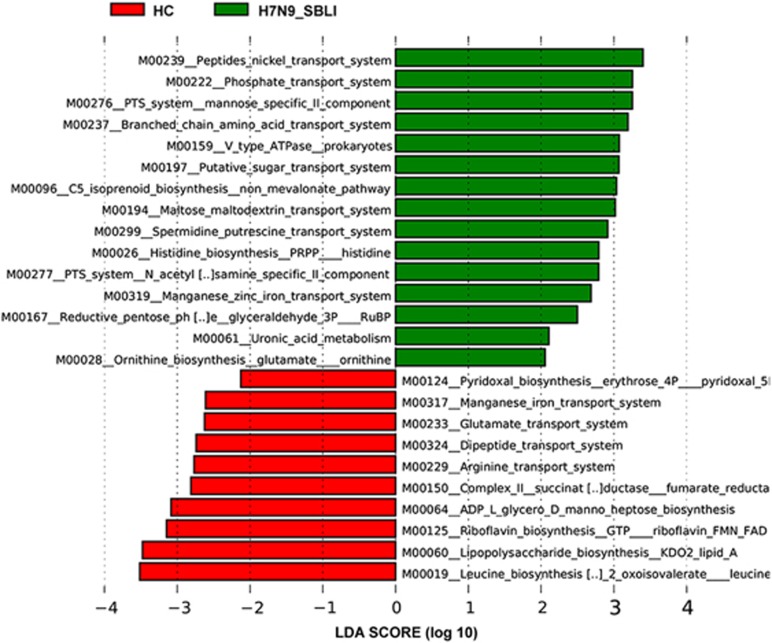
Linear discriminant analysis (LDA) scores were used to predict gene function associated with oropharyngeal (OP) microbiomes in H7N9-infected patients with secondary bacterial lung infection (H7N9_SBLI) using phylogenetic investigation of communities by reconstruction of unobserved states software. OP microbiomes of H7N9 patients with SBLI, SBLI (green); OP microbiomes of healthy controls, HC (red).

PICRUSt software was used to predict the abundance of individual gene families in the three microbiomes. The LEfSe outputs showed that the H7N9_SBLI microbiomes were characterised by an increased number of metabolic pathways related to transporter systems, including phosphate, oligosaccharides, polyol, lipids, minerals and organic ions. In contrast, genes related to vitamin (ridoxal and riboflavin) biosynthesis, leucine biosynthesis, energy metabolism (including ATP and GTP biosynthesis) and lipopolysaccharide biosynthesis were abundant in the HC microbiomes (Figure 7). Specific microbial metabolic pathways could also be used to differentiate between the H7N9_SBLI and H7N9 groups (Supplementary Figure S2). These differences were likely the result of the active metabolism of the H7N9_SBLI OP microbiomes. Metabolic disorder was also observed in the OP microbiomes of the H7N9 group (Supplementary Figure S3).

## DISCUSSION

We monitored SBLI and antibiotic use in all patients in the present study during their hospitalisation and treatment in H7N9 isolation wards using information obtained from medical records and laboratory information systems. Invasive airway surgery and antibiotic administration can damage the defensive functions of the normal respiratory tract microbiome, and highly influence the composition of the OP microbiota, resulting in SBLI. We therefore eliminated 10 OP swap samples from the study because the patients either developed a SBLI during artificial respiration intubation treatment or received empiric prophylactic antibiotic treatment as a result of clinical manifestations of infection, such as fever, sweats, etc. Exclusionary criteria also included factors that may affect the OP or airway microbiome, such as oral disease,^21^ smoking, alcohol abuse,^22^ schizophrenia^23^ and chronic respiratory diseases,^24^ including asthmatic bronchitis and chronic obstructive pulmonary disease. Our aim was to identify any links between the OP microbiome and SBLI.

Dysbiosis of a microbiome (including airway and gut microbiomes) is commonly linked to increased risk and severity of many respiratory diseases.^25^ Although the gut microbiome of H7N9-infected patients has been studied extensively using next-generation sequencing,^3, 26, 27^ little is known about alterations to the microbiome composition in the airways of these patients. In healthy individuals, normal airway bacteria prevent colonisation by invading organisms in a variety of ways, including maintaining an inhospitable local pH, producing bacteriocins, providing a mechanical barrier, interacting with host physiological processes or modulating the host immune response. Microbiota restoration treatment has been shown to reduce the incidence of enterogenous secondary infection, but not exogenous respiratory infection.^3^ Microbiological sampling of the lower respiratory tract is challenging and ethically unfeasible; however, the OP microbiome is more similar to that of the lower airway than the nasopharynx.^28^ Therefore, to understand the relationship between taxonomic alterations in the OP microbiome and SBLI susceptibility, we characterised the OP microbiome of H7N9_SBLI patients using 16S rRNA gene-based sequencing, and compared this with the microbiomes of both H7N9 patients and a HC group.

Increased OP microbiome diversity was detected in both patient groups compared with the HC group, and the difference was significant for the H7N9_SBLI group. This suggested that avian influenza H7N9 virus enhances the adherence of bacteria to OP epithelial cells, thereby increasing the susceptibility of H7N9 patients to SBLI. This association between higher OP microbiome diversity and infection has also been demonstrated in previous studies.^11^ The OP microbiome can be regarded as a reservoir of opportunistic pathogens, and any disturbance of the microbiome may predispose the host to airway infections. In the present study, taxa including Bacteriodaceae, Fusobacteriaceae, Leptotrichiaceae and Pseudomonadaceae were enriched in the OP microbiomes of the H7N9 and H7N9_SBLI groups compared with the HC group. In addition, other taxa such as Actinomycetaceae, Micrococcaceae, Cyanobacteria, Streptococcaceae, Eubacteriaceae, Lachnospiraceae, Ruminococcaceae, Erysipelotrichaceae and Rhizobiaceae were also enriched in the H7N9_SBLI group, suggesting that the greater the OP microbiomedysbiosis, the higher the microbiota diversity, and the more susceptible a host is to SBLI.

The Gammaproteobacteria comprise several medically important groups of bacteria, such as the Enterobacteriaceae, Vibrionaceae and Pseudomonadaceae. Interestingly, most Gammaproteobacteria, especially *Haemophilus* species, were enriched in the HC microbiomes, with the exception of the genus *Pseudomonas*. However, *Pseudomonas* species, which are important aetiological agents of infectious diseases in patients with lung cancer,^29^ were enriched in both patient groups. *Haemophilus* and *Pseudomonas* species likely have both cooperative and antagonistic relationships within the OP microbiome, suggesting that infection is associated with polymicrobial interactions on mucosal surfaces that include commensal bacteria and exogenous pathogens.^30, 31^ *Haemophilus* species, which belong to the Pasteurellaceae family, are always present in low abundance in a healthy adult mucosal microbiome.^32, 33, 34^ This suggests the presence of crucial host physiological functions associated with preventing the establishment of potential pathogens, maintaining the local immune response and keeping the mucosal microbiota balanced.^35^ Immune priming by lipopolysaccharide-mediated Toll-like receptor 4 activation of innate immune cells inhibited influenza virus infection,^36^ while lipopolysaccharide signalling of commensal bacteria contributes to an adequate host innate immune response to viral invasion.^6^

Although the present study did not find any consistently significant differences between the OP microbiomes of the HC and H7N9 groups, OP microbiome LEfSe results revealed that an overwhelming abundance of the genera *Bacteroides*, *Megasphaera*, *Leptotrichia* and *Filifactor* may be associated with avian H7N9 virus infection. These genera are part of the normal microbiota of the oral cavity, and contribute to host immune defences such as activation of the T-cell-dependent immune response and stimulation of antibiotic Paneth cell protein production.^37^ Some species, such as *Bacillus fragilis*,^37^ *Leptotrichia* sp.^38^ and *Filifactoralocis*,^39^ are opportunistic human pathogens capable of causing oral infections. A high relative abundance of *Megasphaera* sp. and *Leptotrichia* sp. is thought to help maintain a healthy local acidic environment in the vagina of bacterial vaginosis patients as a compensatory measure against the low abundance of *Lactobacillus* species.^40, 41^ However, enrichment of these lactic acid-producing bacteria in the OP could increase the risk of influenza virus infection because the low-pH environment generated by these species could trigger the fusion of the viral and cellular membranes.^42^ In the present study, overgrowth of lactic acid-producing bacteria like *Megasphaera* and *Leptotrichia* species in OP samples was related to susceptibility to H7N9 infection.

It has long been suspected that viral infections of the respiratory tract predispose the host to bacterial superinfections through the disruption of the respiratory mucosal epithelium, thereby promoting bacterial adhesion to respiratory epithelial cells.^43^ When compared with the HC group, the degree of OP microbiota dysbiosis in the H7N9_SBLI group was more severe than in the H7N9 group. It is likely that the primary virus infection disturbed the OP microbiota, which increased the host susceptibility to SBLI, which in turn further aggravated the dysbiosis of the OP microbiome. *Leptotrichia*, *Oribacterium*, *Streptococcus*, *Atopobium*, *Eubacterium*, *Solobacterium* and *Rothia* were the key genera associated with SBLI. Most of these genera have been detected in low abundance in healthy OP microbiomes.^44^ For example, fluctuations in the endogenous opportunistic pathogens in the OP microbiota can cause infections of the airways.^11^ In the present study, we found that opportunistic pathogenic genera, including *Streptococcus*, *Actinomyces*, *Rothia*, *Eubacterium*, *Oribacterium* and *Mogibacterium*, were more abundant in the OP microbiomes of H7N9_SBLI patients than that of H7N9 patients. Of these bacteria, *Actinomyces*, *Streptococcus* and *Eubacterium* species were found to colonise the lower airways of lung cancer patients,^44^ while *Streptococcus*, *Rothia* and *Atopobium* species are associated with caries progression.^45, 46^ *Rothia* species are also thought to contribute to the pathogenesis of pneumonia, particularly in immunocompromised hosts.^8^ Furthermore, the presence and density of *Rothia* in the upper respiratory tract has been linked to an increased risk of otitis media in children.^47^ *Mogibacterium* and *Oribacterium* are enriched in the sputum microbiota of tuberculosis patients.^48^ In the present study, *Eubacterium*, *Rothia*, *Streptococcus* and *Oribacterium*, which showed a >100-fold difference in relative abundance between the H7N9_SBLI and H7N9 groups, are likely to be associated with susceptibility to SBLI in patients with avian H7N9 virus infection.

Unfortunately, this study couldn’t illustrate whether shifts in the microbial community structures were H7N9-infection-specific. It is a challenge for us to enrol matched subjects with other significant respiratory viral pathogen infections. In addition, most subjects with non-H7N9 infections were excluded in our study, as the pathogens were very diverse and not identified by routine work. Future study on OP microbiome in cohorts with other significant respiratory pathogen infections will help to determine the key bacterium specifically associated with H7N9 infections.

In summary, we identified aberrant OP microbial communities in H7N9 patients, with the OP microbiome of H7N9_SBLI patients characteristically dominated by several key pathogenic airway microorganisms. Microbiome perturbations may be critical for disease risk. Current studies focus on describing the variant microbe populations those appear in the disease state or temporal microbial changes over the course of SBLI. We hypothesise that immune dysfunction in H7N9 patients might promote the growth of disease-associated OP bacteria, and subsequently cause dysbiosis of OP microbiota and enhanced susceptibility to infection by pathogens and facultative bacteria. For many conditions, the challenge is to discover whether there is a causal link between microbiome variation and significant pathology. Animal studies and other models to elucidate the molecular mechanisms of the observed species in relation to increased risk of developing SBLI are suggested to perform in the future.

## Figures and Tables

**Table 1:** Clinical characteristics of all subjects

**Characteristics**	**Secondary infection group (H7N9_SBLI,** ***n*****=21)**	**Non-secondary infection group (H7N9,** ***n*****=30)**	**HCs (*****n*****=30)**
Age, mean (years)	60.5 (13.5)	53 (12.7)	50 (9.3)
Male:female	12:9	17:13	11:19
WBC (10^9^ cells/L)	8.5 (3.6)*^,Δ^	3.6 (2.5)	4.1 (2.2)
CRP (mg/L)	97.9 (56.7)*^,Δ^	44.6 (47.3)	No detection

*Infection with*			
*Klebsiella pneumoniae* (IFS, n)	6	0	0
*Acinetobacter baumanii* (IFS, n)	7	0	0
*Pseudomonas* (IFS, n)	11	0	0

*Co-infection with*			
*Flavobacterium* indologenes (IFB, n)	2	0	0
*Staphylococcus* (IFB, n)	4	0	0
* Candida albicans* (IFS, n)	9	0	0

Abbreviations: C-reaction protein, CRP; isolated from blood, IFB, isolated from sputum, IFS; white blood cell, WBC.

*P<0.05 (comparison between H7N9 and H7N9_SBLI patient groups); ^Δ^*P*<0.05 (comparison between the H7N9_SBLI and HC groups).

Age, WBC counts and CRP levels are shown as the mean (sd).
