# Supplementary material for: Disordered oropharyngeal microbial communities in H7N9 patients with or without secondary bacterial lung infection
Source: Emerg Microbes Infect. 2017 Dec 20;6(12):e112–. doi: 10.1038/emi.2017.101 (PMC5750457; doi:10.1038/emi.2017.101)

**Supplementary Figure S1** Bacterial diversity clustering by combining unweighted and weighted UniFrac PCoA of OP microbiota. (A) Unweighted UniFrac (qualitative); (B) Weighted UniFrac (qualitative). Each symbol represents a sample (blue, HC; green, H7N9; and red, H7N9_SBLI); the variance explained by the PCs is indicated in parentheses on the axes. H7N9, OP microbiome of patients with H7N9 virus infection; H7N9_SBLI, OP microbiome of H7N9 patients with SBLI; HC, healthy control OP microbiome.


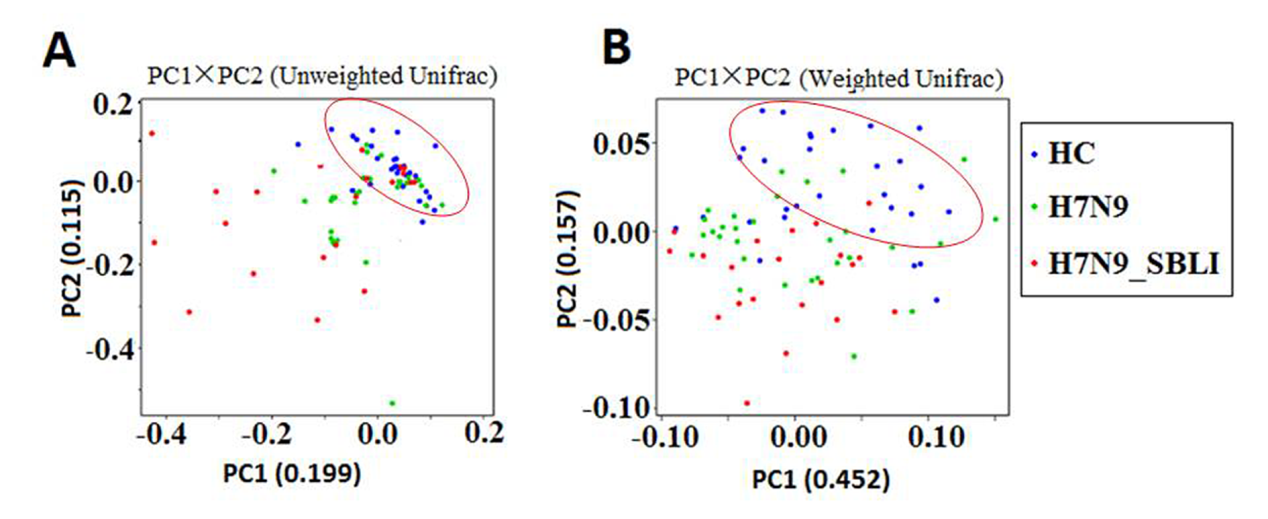

Supplement: Supplementary Figure S1 [file emi2017101x1.docx]
