# Supplementary material for: Disordered oropharyngeal microbial communities in H7N9 patients with or without secondary bacterial lung infection
Source: Emerg Microbes Infect. 2017 Dec 20;6(12):e112–. doi: 10.1038/emi.2017.101 (PMC5750457; doi:10.1038/emi.2017.101)

**Supplementary Figure S3** LDA scores predict gene function associated with OP microbiomes in H7N9_SBLI patients when compared to H7N9 patients using PICRUSt. PICRUSt: Phylogenetic Investigation of Communities by Reconstruction of Unobserved States. H7N9 (red), OP microbiome of H7N9 patients; SBLI (green), OP microbiome of H7N9 patients with SBLI.


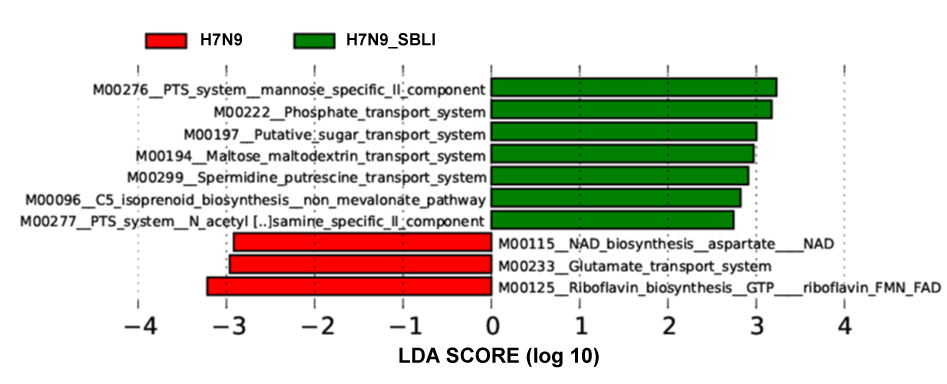

Supplement: Supplementary Figure S3 [file emi2017101x3.docx]
