# Supplementary material for: Disordered oropharyngeal microbial communities in H7N9 patients with or without secondary bacterial lung infection
Source: Emerg Microbes Infect. 2017 Dec 20;6(12):e112–. doi: 10.1038/emi.2017.101 (PMC5750457; doi:10.1038/emi.2017.101)
Supplement: Supplementary Table S1 [file emi2017101x4.docx]

**Supplementary Table S1** Questionnaire: data for subjects participating in the investigation of the OP microbiome

**Data for subjects participated in the investigation of the OP swab microbiome**

**Name： sex：**F/M **Date of birth (**month-year**)：**

**Telephone number(s)： Sample NO:**

| Data of follow-up(Y-M-D) | | |  | | | |
| --- | --- | --- | --- | --- | --- | --- |
| Way of follow- up | | | 1. outpatient 2 inpatient | | | |
| A survey   of disease symptom | 1.Upper and lower airway , lung,oral or previous related surgery  2.Basic diseases such as diabetes, coronary disease,hypertension  3. Ascites,or gastrointestinal bleeding  4.  [asthma](http://dict.youdao.com/w/asthma/#keyfrom=E2Ctranslation), or Chronic airway inflammation  5. pulmonary fibrosis or  lung carcinoma  6.Co-infection with HCV, HIV or other secondary bacterial infection  7.Symptoms of respiratory: shortness of breath, sputum production,wheezing or chest pain or discomfort.  8.Oothers | |  | | | |
| X-ray examation | | | Examation day： | | | |
|  |  |  |  | | | |
| Oral Health Screening | | | Examation day： | | | |
|  |  |  | 1. [periodontitis](http://dict.youdao.com/w/periodontitis/) □ 2. oral ulcer □ 3. Cavities □ 4. others: ______ | | | |
| Biochemical blood routine inspection | | | Examation day： | | | |
|  |  |  | 1. positive 2. negative | | | |
| \| Rapid test for influenza A \| Examation day： \| \| --- \| --- \| \|  \| | | | | | | |
| \| Nucleic acid (H7N9) PCR testing \| Examation day： \| \| --- \| --- \| \| 1. positive 2. negative \| | | | | | | |
| Physical sign | Blood pressure（mmHg） | |  | | | |
|  | Weight（kg） | |  | | | |
|  | [Height](javascript:void(0);) (cm) | |  | | | |
|  | Body mass index （BMI） | |  | | | |
|  | Others | |  | | | |
| Lifestyles | Daily consumption of cigarettes | |  | | | |
|  | Daily consumption of  alcohol | |  | | | |
|  | Others (including food habits and other drugs) | |  | | | |
|  | Fitness habits | | Form: Time: | | | |
|  | Dietaryhabits | Yoghurt |  | | | |
|  |  | staple |  | | | |
|  | Psychological quality | | 1.better 2.good 3.worse □ | | | |
|  | Treatment compliance | | 1.better 2.good 3.worse □ | | | |
| Drugs | Antibiotics | |  | | | |
|  | Probiotics | |  | | | |
|  | Or others | |  | | | |
|  | Drugs 1: (name) | |  | | | |
|  | Drug dosage | | Times/Day: Dosage/time: mg | | |  |
|  | Drugs 2:(name) | |  |  |  | |
|  | Drug dosage | | Times/Day: Dosage/time: mg | | |  |
|  | Drugs 3:(name) | |  | | | |
|  | Drug dosage | | Times/Day: Dosage/time: mg | | |  |
|  | Others | |  | | | |
| Other need to supplement during hospitalization and treatment (SBLI, artificial respiration intubation surgery or antibiotics treatment ) | | |  | | | |
| Patients signature | | |  | | | |
| Doctor signature | | |  | | | |
