# Supplementary material for: Disordered oropharyngeal microbial communities in H7N9 patients with or without secondary bacterial lung infection
Source: Emerg Microbes Infect. 2017 Dec 20;6(12):e112–. doi: 10.1038/emi.2017.101 (PMC5750457; doi:10.1038/emi.2017.101)
Supplement: Supplementary Table S2 [file emi2017101x5.docx]

| **Supplementary Table S2** *P*-values for stastical anaylsis of predominant phyla in OP microbiome between groups using the nonparametric Mann-Whitney U test. | | | |
| --- | --- | --- | --- |
|  |  |  |  |
|  |  |  |  |
|  | P-values ( H7N9_SBLI and HCs) | P-values( H7N9 and HCs) | P-values (H7N9 and H7N9_SBLI) |
| p__Bacteroidetes | 0.013 | 0.16 | 0.31 |
| p__Proteobacteria | 0.085 | 0.107 | 0.745 |
| p__Firmicutes | 0.005 | 0.225 | 0.108 |
| p__Fusobacteria | 0.008 | 0.003 | 0.789 |
| p__Candidate_division_TM7 | 0.605 | 0.478 | 0.32 |
| p__Actinobacteria | 0.001 | 0.469 | 0.003 |
| p__Spirochaetae | 0.491 | 0.038 | 0.444 |
| p__Tenericutes | 0.646 | 0.261 | 0.2 |
| p__Synergistetes | 0.413 | 0.278 | 0.785 |
| p__BD1-5 | 0.025 | 0.137 | 0.002 |
| p__Candidate_division_SR1 | 0.969 | 0.882 | 0.745 |
